# Multimodal Recruitment to Study Ovulation and Menstruation Health: Internet-Based Survey Pilot Study

**DOI:** 10.2196/24716

**Published:** 2021-04-16

**Authors:** Shruthi Mahalingaiah, J Jojo Cheng, Michael R Winter, Erika Rodriguez, Victoria Fruh, Anna Williams, MyMy Nguyen, Rashmi Madhavan, Pascaline Karanja, Jill MacRae, Sai Charan Konanki, Kevin J Lane, Ann Aschengrau

**Affiliations:** 1 Department of Environmental Health Harvard T.H. Chan School of Public Health Boston, MA United States; 2 Department of Obstetrics and Gynecology Boston University School of Medicine Boston, MA United States; 3 Department of Epidemiology Boston University School of Public Health Boston, MA United States; 4 Department of Biostatistics and Medical Informatics University of Wisconsin Madison, WI United States; 5 Biostatistics and Epidemiology Data Analytics Center Boston University School of Public Health Boston, MA United States; 6 Department of Environmental Health Boston University School of Public Health Boston, MA United States

**Keywords:** polycystic ovary syndrome, PCOS, menstrual cycle, multimodal recruitment strategy, epidemiology, recruitment, pilot, strategy, women, feasibility, online survey, ovulation, menstrual

## Abstract

**Background:**

Multimodal recruitment strategies are a novel way to increase diversity in research populations. However, these methods have not been previously applied to understanding the prevalence of menstrual disorders such as polycystic ovary syndrome.

**Objective:**

The purpose of this study was to test the feasibility of recruiting a diverse cohort to complete a web-based survey on ovulation and menstruation health.

**Methods:**

We conducted the Ovulation and Menstruation Health Pilot Study using a REDCap web-based survey platform. We recruited 200 women from a clinical population, a community fair, and the internet.

**Results:**

We recruited 438 women over 29 weeks between September 2017 and March 2018. After consent and eligibility determination, 345 enrolled, 278 started (clinic: n=43; community fair: n=61; internet: n=174), and 247 completed (clinic: n=28; community fair: n=60; internet: n=159) the survey. Among all participants, the median age was 25.0 (SD 6.0) years, mean BMI was 26.1 kg/m^2^ (SD 6.6), 79.7% (216/271) had a college degree or higher, and 14.6% (37/254) reported a physician diagnosis of polycystic ovary syndrome. Race and ethnicity distributions were 64.7% (176/272) White, 11.8% (32/272) Black/African American, 7.7% (21/272) Latina/Hispanic, and 5.9% (16/272) Asian individuals; 9.9% (27/272) reported more than one race or ethnicity. The highest enrollment of Black/African American individuals was in clinic (17/42, 40.5%) compared to 1.6% (1/61) in the community fair and 8.3% (14/169) using the internet. Survey completion rates were highest among those who were recruited from the internet (159/174, 91.4%) and community fairs (60/61, 98.4%) compared to those recruited in clinic (28/43, 65.1%).

**Conclusions:**

Multimodal recruitment achieved target recruitment in a short time period and established a racially diverse cohort to study ovulation and menstruation health. There were greater enrollment and completion rates among those recruited via the internet and community fair.

## Introduction

Polycystic ovary syndrome, initially described in 1935 [1], is now considered the most common endocrine disorder in reproductive-aged women [2]. Affecting 10% to 15% [2], prevalence estimates range from as low as 8% [3] to as high as 26% [4], depending on the definition used and population studied. The disorder is characterized by clinical or biochemical androgen excess, menstrual irregularity, and the presence of polycystic ovarian morphology on ultrasound visualization [5]. Clinical androgen excess typically presents as acne, hirsutism, or androgenic alopecia [6-8]. Women with polycystic ovary syndrome may also experience infertility, insulin resistance, and obesity [9-12].

Existing research on polycystic ovary syndrome is typically conducted in either small clinical cohorts or larger epidemiologic studies. The latter have variable ascertainment of the disease or disease features which may increase misclassification of disease state and bias estimates of risk [13]. Preliminary studies on polycystic ovary syndrome in existing population-based cohorts such as the Nurses’ Health Study 2, the Framingham Heart Study, and the Cape Cod Health Study were limited by poor correlation of polycystic ovary syndrome self-reports compared to medical records [14], too few reported cases of menstrual irregularity [15], and inadequate determination of phenotype [16], respectively. Inaccurate classification using single yes or no questions to identify women with polycystic ovary syndrome may bias estimates of the disease.

Existing epidemiologic cohorts were also limited with respect to race and ethnic diversity; more than 90% of participants were White in these 3 cohorts [16-18], thereby limiting the ability to detect differences in prevalence and etiological associations across racial and ethnic groups. Racial and ethnic-specific differences in androgen excess and metabolic syndrome symptoms are well established but the reasons for this are not well understood [19-21].

The Ovulation and Menstruation Health Pilot Study was conducted to (1) determine the feasibility of enrolling participants from diverse backgrounds using varied recruitment modalities, and (2) understand how survey completion status differed by participant characteristics.

## Methods

### Design

The Ovulation and Menstruation Health study website consists of a short animated educational recruitment video, a web-based consent form, screening questions, and a survey instrument. The animated video was designed to appeal to a diverse audience, as its illustrations included women of all races and body types. The goal of the Ovulation and Menstruation Health Pilot Study was to recruit at least 200 reproductive-aged women over a 1-year period. Those who were menstruating or had the capacity to menstruate were eligible (trans-male and other). Individuals who were <18 years or >45 years of age; identified as male; were pregnant at the time of the survey; had a hysterectomy/oophorectomy, amenorrhea due to radiation, or chemotherapy; or were unwilling or unable to provide an email address were deemed ineligible. The survey instrument was publicly accessible from August 21, 2017 to February 26, 2018 on the study website and was used in each recruitment modality. The Boston Medical Center and Boston University Medical Campus Institutional Review Boards approved the study (IRB H-35075).

### Incentive

The first 200 participants who completed the survey were entered into a lottery for a US $200 gift card. All who were approached for recruitment were offered free earphones.

### Recruitment

The multimodal recruitment locations were in clinic, a Boston-based community event, and the internet. The recruitment approach was adapted to meet the needs of each recruitment location.

#### In-Clinic

In-clinic recruitment occurred from September to October 2017. Approximately 2000 informational recruitment letters including the study website were sent during this recruitment period to patients with an upcoming visit to the Department of Obstetrics and Gynecology (OB/GYN) at Boston Medical Center (BMC). For 20 days, research assistants showed the study website and the promotional video via an electronic tablet to interested persons at 2 OB/GYN waiting rooms.

#### Community Event

A recruitment table was set up at the Boston Women’s Market in the Jamaica Plain neighborhood of Boston on September 17, 2017. This community event was an opportunity for those who identify as female or supporters of women’s causes to sell products from their small businesses.

#### Internet

Internet-based recruitment methods included sending out email communications, creating study social media engagement accounts, and sharing recruitment materials on individual social networks of the study staff. The study website and social media pages were discoverable on any internet search. Boston University Medical Campus (BUMC)–wide electronic communications, which included a brief description of the study, eligibility requirements, incentive, and study contact information, began in September 2017 and ran for the duration of the enrollment period. The study website link was shared on personal networks of study personnel (RM, MN, AW) with the Boston University Masters of Medical Science Class of 2018 and the Bates Feminist Collective Facebook groups in September 2017.

Physical flyers and business cards (paper materials) were posted around the BUMC campus for the duration of the study, and at Boston Skin Solutions, a center that treats excess facial and body hair on September 27, 2017.

### Informed Consent, Screening, and Survey Initiation

The web-based consent form included questions on a participant’s interest in follow-up surveys, other studies, and contributing biospecimens. Once consented, the individual was presented with an eligibility screener. Eligible participants recruited in clinic were able to start the survey in the waiting room. For eligible participants from the community event and the internet, a unique link to the survey was emailed to the participant’s email address. For all enrolled participants, up to 3 reminders were sent on consecutive days to those who did not complete the survey.

### Survey Description

The Ovulation and Menstruation Health Pilot Study survey had a total of 10 sections, with 219 questions; however, most respondents had fewer questions due to skip patterns. The About You section included questions on current residence and whether they ever received care at BMC. The Demographics section included questions on race and ethnicity, education, birth country, and income. The Anthropometrics section captured data regarding height, weight, and body shape using a pictorial tool. The Menstrual Cycle section asked about menarche, regularity of cycles, and cycle tracking. The Hormone Usage section obtained data on hormonal contraceptive usage, history and reasons for hormone usage. The Health and Body section included questions for self-reported androgen excess using a pictorial tool for androgenic alopecia and hirsutism based on the modified Ferriman-Gallwey scale [22]; polycystic ovary syndrome status (questions specific to polycystic ovary syndrome diagnosis, symptoms for diagnosis, and medication and supplement use, and family history of polycystic ovary syndrome); reproductive health (history of infertility, uterine fibroids, endometriosis, and premature ovarian failure); general health (diagnosed medical conditions such as hypertension, diabetes, and other chronic conditions and their treatment); diet and lifestyle (limited questions about alcoholic, nonalcoholic beverage consumption, and smoking habits); obstetric history (ever pregnant, number of prior pregnancies, and each pregnancy outcome, including stillbirths and miscarriages, and live births). For every livebirth, an additional question was asked regarding gestational length and birth outcomes such as birth weight. Study data were recorded and stored on encrypted servers at the Boston University School of Public Health Biostatistics & Epidemiology Data Analytics Center. The Ovulation and Menstruation Health Pilot Study survey can be found on the Harvard Dataverse [23].

### Data Analysis

Participant characteristics were evaluated by recruitment location and survey completion. Those who started the survey via the internet included those who input the link into their web-browser after learning of the study from any web-based or printed recruitment materials and completed the web-based consent and screener. Survey initiation was defined as anyone who started the “About You” section. Survey completion was defined as having completed the first question of the “Pregnancy and Birth History” upon entering the total number of pregnancies experienced. Those who entered 0 pregnancies had no further questions to complete. Those who entered 1 or more had additional pregnancy related questions to complete. For both nulliparous and multiparous responses, completing the survey was as entering the total number of pregnancies experienced. To assess the geographical catchment area associated with the survey, participants’ self-reported home states were mapped.

## Results

### Recruitment and Enrollment

**Figure 1:**
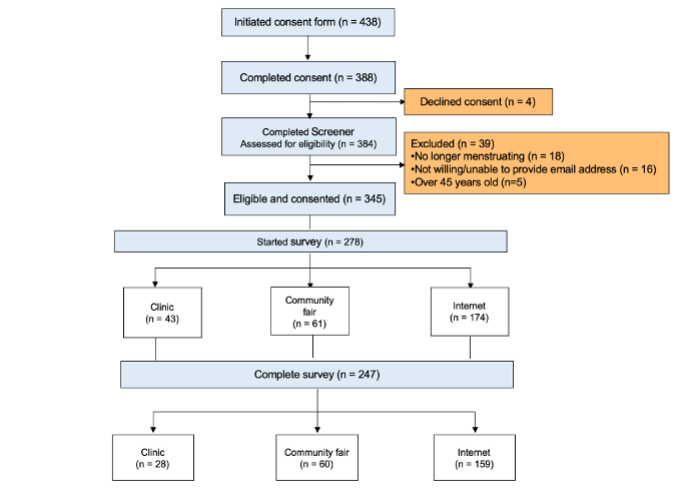
Flowchart of participants enrolled in the Ovulation and Menstruation Health Pilot Study.

**Figure 2:**
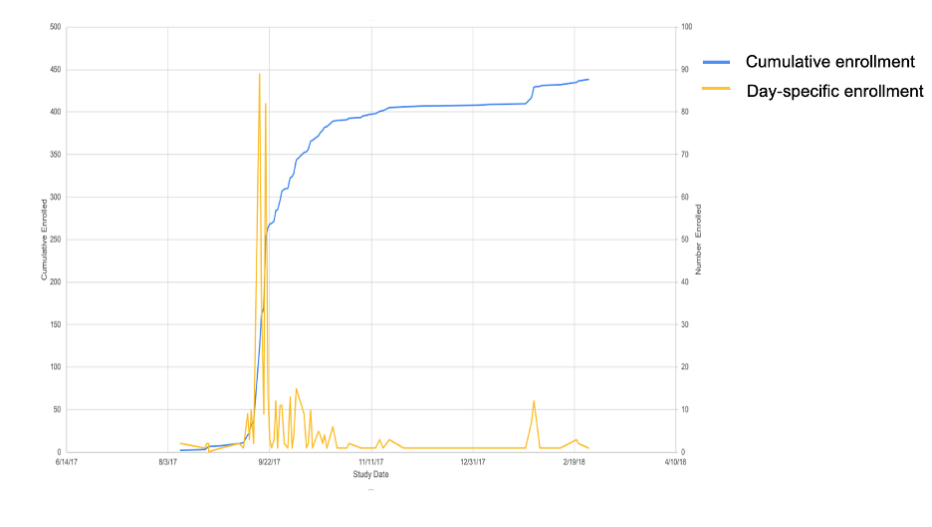
Ovulation and Menstruation Health Pilot Study enrollment graph.

Recruitment exceeded our target of 200 women in 8 weeks. During the 6-month recruitment window, 438 women began the consent process, 384 were assessed for eligibility, and 345 women were screened and included (88.7% of those assessed for eligibility). Of the 345, 278 women started the survey, of whom 66 were recruited in clinic, 61 were recruited from the community event, and 174 were recruited from the internet. Figure 1 displays the study enrollment flow chart. Spikes in cumulative enrollment coincided with the active in-clinic recruitment and the community event (Figure 2). Out of the 384 participants who consented and completed screening, only 39 (10.2%) were excluded. Of the individuals who were excluded, 18 were no longer menstruating, 16 were unwilling or unable to provide email addresses, and 5 were over the age of 45 years. Among those who initiated the survey and responded to re-contact questions, agreement for re-contact was high—96.0% (266/277) consented to be re-contacted for additional information, 81.2% (225/277) for biological samples, 96.4% (267/277) for a follow-up survey, and 79.4% (220/277) for a different research study.

### Participant Characteristics

**Figure 3:**
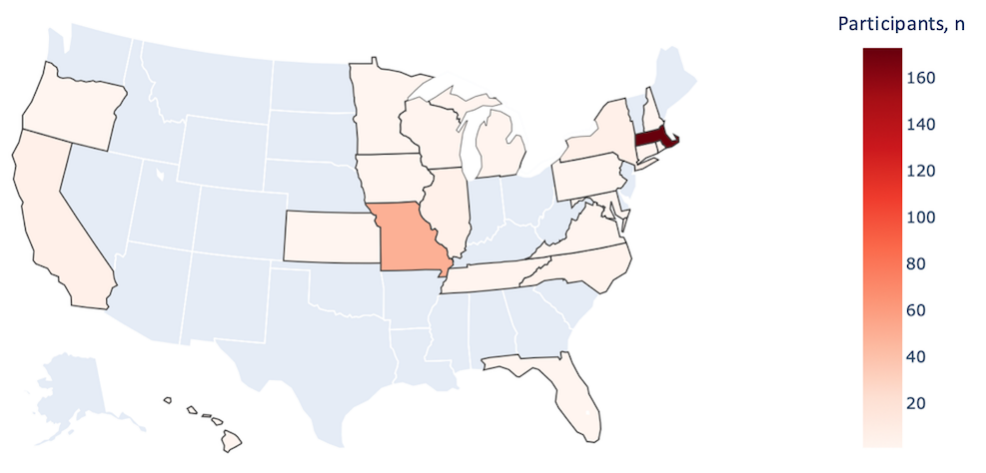
Ovulation and Menstruation Health Study participant population map.

Among the 278 women who started the survey, the average age was 27.3 years and the median age was 25.0 (SD 6.0), with the majority (224/273, 82.1%) born in the United States (Table 1). Participants were White (176/272, 64.7%), Black/African American (32/272, 11.8%), Latina/Hispanic (21/272, 7.7%), and mixed race (27/272, 9.9%). Two individuals identified their gender as other (nonbinary or gender queer). While the majority (216/271, 79.7%) had a 4-year college degree or more, 7.0% (19/271) had a high school degree or less education. Participants were distributed across all income categories, with 21.3% (58/272) in the less than $25,000 category. The mean BMI was 26.1 kg/m^2^ (SD 6.6), with 15.4% (41/266) in the overweight category; 13.1% (32/245) had smoked at least 100 cigarettes over their lifetime. The reported prevalence of doctor-diagnosed polycystic ovary syndrome was 14.6% (37/254), and 82.3% (218/265) had ever used hormonal contraception. The geographic distribution of the cohort included 20 states (Figure 3). The majority of the participants were recruited from Massachusetts (n=173) and Missouri (n=50).

**Table 1:** Demographics in the Ovulation and Menstruation pilot study.

Characteristic		Value (n=278^a^)
Age (years), median (SD)		25.0 (6.0)
Born in the United States, n (%)		224 (82.1)
**Race, n (%) **		
	White	176 (64.7)
	Latina/Hispanic	21 (7.7)
	Black/African American	32 (11.8)
	Asian	16 (5.9)
	More than one race	27 (9.9)
**Educational attainment, n (%) **		
	High school graduate/GED or less education	19 (7.0)
	Some college or 2-year degree	36 (13.3)
	4-year college graduate	101 (37.3)
	More than 4-year college degree	115 (42.4)
**Total annual household income, n (%) **		
	Below $25,000	58 (21.3)
	$25,000 to $49,999	70 (25.7)
	$50,000 to $74,999	44 (16.2)
	$75,000 to $99,999	20 (7.4)
	$100,000 or more	43 (15.8)
	Prefer not to answer	17 (6.3)
	Don't know	20 (7.4)
Smoked at least 100 cigarettes over lifetime, n (%)		32 (13.1)
**BMI^b^, n (%) **		
	Underweight (<18.5 kg/m^2^)	8 (3.0)
	Normal weight (18.5-24.9 kg/m^2^)	153 (57.5)
	Overweight (25.0-29.9 kg/m^2^)	41 (15.4)
	Obese (≥30.0 kg/m^2^)	64 (24.1)
Polycystic ovary syndrome diagnosis by doctor, n (%)		37 (14.6)
Ever pregnant, n (%)		42 (17.0)
**Gravidity^c^, n (%) **		
	1 pregnancy	17 (40.5)
	2 pregnancies	10 (23.8)
	3 pregnancies	7 (16.7)
	>4 pregnancies	8 (19.1)
Hormonal contraceptives ever use, n (%)		218 (82.3)
**Self-rated current health, n (%) **		
	Excellent	37 (14.1)
	Very good	108 (41.2)
	Good	91 (34.7)
	Fair	25 (9.5)
	Poor	1 (0.4)
Survey completed, n (%)		247 (88.9)

^a^Missing—born in the United States n=5 (1.8%); race: n=6 (2.2%); education: n=7 (2.5%); income: n=6 (2.2%); smoking status: n=33 (11.9%); BMI: n=12 (4.3%); polycystic ovary syndrome diagnosis: n=24 (8.6%); gravidity: n=26 (9.4%); ever pregnant: n=31 (11.2%); hormonal contraceptive: n=13 (4.7%); self-rated current health: n=16 (5.8%).

^b^BMI: body mass index.

^c^Gravidity among women who were ever pregnant (n=42).

### Population Demographic Characteristics by Recruitment Location

Differences were found in age, birth country, race, educational attainment, BMI, and physician-diagnosed polycystic ovary syndrome, by location of recruitment (Table 2). Women recruited in clinic tended to be older (median 34.0 years, SD 6.7), born outside the US (26/42, 61.9%), and with lower educational attainment, 19.0% (8/42) were high school graduate/GED or less education compared to women recruited at the community fair and via the internet. Women recruited in clinic were also more likely to be obese (18/38, 47.4%), have had a physician diagnosis of polycystic ovary syndrome (12/33, 36.4%), and less likely to have ever used oral contraceptives (23/37, 62.2%) compared to women recruited at the community fair and via the internet. Those from the community fair and internet were more likely to be born in the US (58/61, 95.1% and 150/170, 88.2%, respectively), White (47/61, 77.0% and 121/169, 71.6%, respectively), and have greater than a 4-year college degree (24/61, 39.3% and 83/168, 49.4%, respectively) than those recruited from the clinic. Women recruited from outside the clinic were younger (community fair: median 23.0, SD 4.0; internet: median 25.0, SD 5.4 years), leaner BMI (community fair: 23.3 kg/m^2^ SD 4.8; internet: 23.5 kg/m^2^ SD 7.0) and had a lower prevalence of physician diagnosed polycystic ovary syndrome (community fair: 3/61, 4.9%; internet: 22/160, 13.8%). Of note, 46.4% of women recruited in clinic (13/28) had been pregnant compared to 3.3% (2/61) and 17.0% (27/159) for the community fair and internet participants, respectively. Income and smoking status were similar among recruitment locations.

**Table 2:** Demographic characteristics by recruitment location.

Characteristic		Location		
		Clinic (n=43)^a^	Community fair (n=61)^b^	Internet (n=174)^c^
Age (years), median (SD)		34.0 (6.7)	23.0 (4.0)	25.0 (5.4)
Born in the United States, n (%)		16 (38.1)	58 (95.1)	150 (88.2)
**Race, n (%)**				
	White	8 (19.0)	47 (77.0)	121 (71.6)
	Latina/Hispanic	12 (28.6)	2 (3.3)	7 (4.1)
	Black/African American	17 (40.5)	1 (1.6)	14 (8.3)
	Asian	3 (7.1)	1 (1.6)	12 (7.1)
	More than one race	2 (4.8)	10 (16.4)	15 (8.9)
**Educational attainment, n (%)**				
	High school graduate/GED or less education	8 (19.0)	6 (9.8)	5 (3.0)
	Some college or 2-year degree	14 (33.3)	8 (13.1)	14 (8.3)
	4-year college graduate	12 (28.6)	23 (37.7)	66 (39.3)
	More than 4-year college degree	8 (19.1)	24 (39.3)	83 (49.4)
**Total annual household income, n (%)**				
	Below $25,000	4 (9.5)	13 (21.3)	41 (24.3)
	$25,000 to $49,999	12 (28.6)	20 (32.8)	38 (22.5)
	$50,000 to $74,999	11 (26.2)	9 (14.8)	24 (14.2)
	$75,000 to $99,999	1 (2.4)	3 (4.9)	16 (9.5)
	$100,000 or more	5 (11.9)	6 (9.8)	32 (18.9)
	Prefer not to answer	8 (19.1)	2 (3.3)	7 (4.1)
	Don't know	1 (2.4)	8 (13.1)	11 (6.5)
Smoked at least 100 cigarettes over lifetime, n (%)		5 (17.9)	5 (8.3)	22 (14.0)
**BMI^d^, n (%)**				
	Normal weight or underweight (≤25 kg/m^2^)	14 (36.8)	40 (65.6)	107 (64.1)
	Overweight (25-30 kg/m^2^)	6 (15.8)	11 (18.0)	24 (14.4)
	Obese (≥30 kg/m^2^)	18 (47.4)	10 (16.4)	36 (21.6)
Polycystic ovary syndrome diagnosis by doctor, n (%)		12 (36.4)	3 (4.9)	22 (13.8)
Ever pregnant, n (%)		13 (46.4)	2 (3.3)	27 (17.0)
**Gravidity^e^, n (%)**				
	1 pregnancy	5 (38.5)	1 (50.0)	11 (40.7)
	2 or more pregnancies	8 (61.5)	1 (50.0)	16 (59.3)
Hormonal contraceptives ever use, n (%)		23 (62.2)	54 (88.5)	141 (84.4)
**Self-rated current health, n (%)**				
	Excellent	6 (16.2)	8 (13.1)	23 (14.0)
	Very good	7 (18.9)	29 (47.5)	72 (43.9)
	Good	18 (48.7)	21 (34.4)	52 (31.7)
	Fair to poor	6 (16.2)	3 (4.9)	17 (10.4)
Survey completed, n (%)		28 (65.1)	60 (98.4)	159 (91.4)

^a^Missing—born in the US: n=1 (2.3%); race: n=1 (2.3%); education: n=1 (2.3%); income: n=1 (2.3%); smoking status: n=15 (34.9%); BMI: n=5 (11.6%); polycystic ovary syndrome diagnosis: n=10 (23.3%); ever pregnant: n=15 (34.9%); hormonal contraceptive: n=6 (14.0%); self-rated current health: n=6 (14.0%).

^b^Missing—smoking status: n=1 (1.6%).

^c^Missing—born in the US: n=4 (2.3%); race: n=5 (2.9%); education: n=6 (3.5%); income: n=5 (2.9%); smoking status n=17 (9.8%); BMI: n=7 (4.0%); polycystic ovary syndrome diagnosis: n=14 (8.1%); ever pregnant: n=15 (8.6%); hormonal contraceptive: n=7 (4.0%); self-rated current health: n=10 (5.7%).

^d^BMI: body mass index.

^e^Gravidity among women who were ever pregnant (n=42).

### Survey Completion

Among the 278 women who started the survey, 247 (88.8%) completed it. The median time to completion was 12.6 minutes (range 1.03 minutes to 92 days); 7 participants took more than 24 hours to complete the survey. Women completing the survey were more likely to be US born (210/246, 85.4% vs 14/27, 51.9%), White (165/245, 67.3% vs 11/27, 40.7%) and have 4 years of college education or more (200/244, 81.9% vs 16/27, 59.2%) compared to those who did not complete the survey (Table 3).

**Figure 4:**
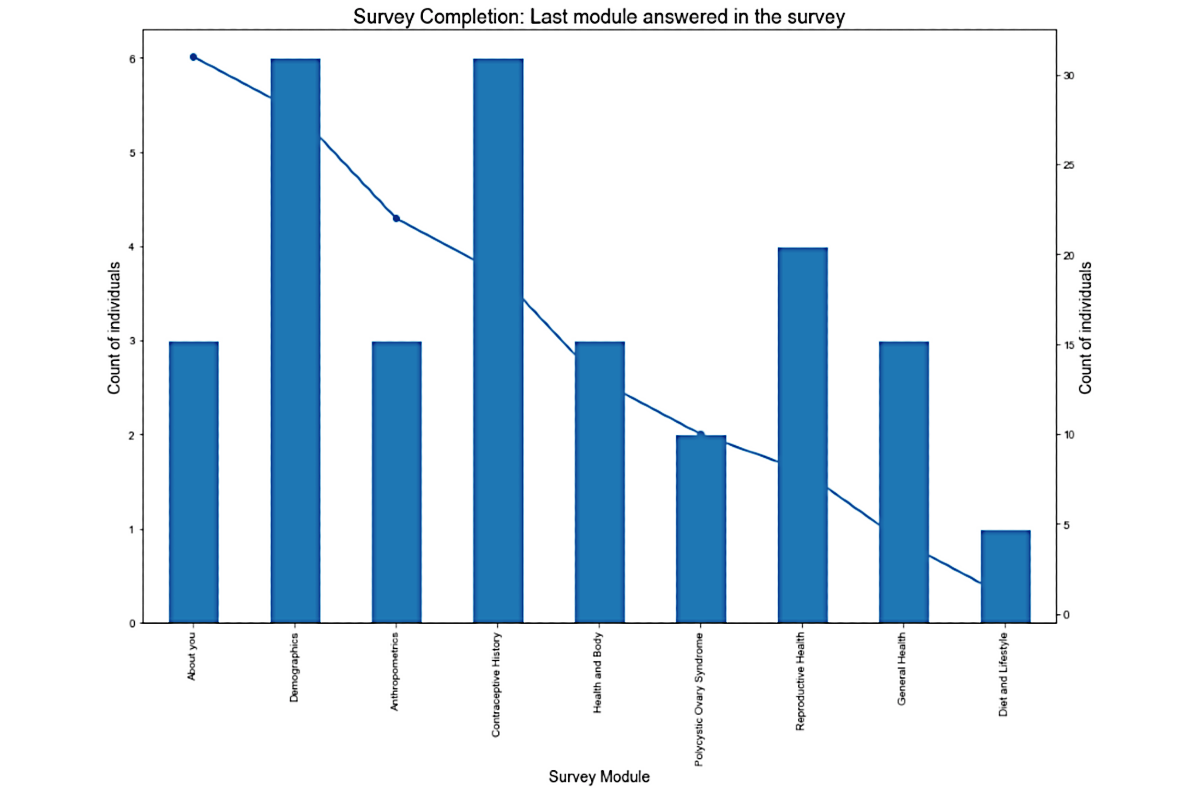
Survey completion by last module answered in the survey.

We also found that the women who did not complete the survey were older and had higher annual household income compared to those who completed the survey. Among the 31 women who did not complete the survey, dropout seemed equally distributed across section categories (Figure 4).

**Table 3:** Demographic characteristics by survey completion status (N=278).

Characteristic		Complete (n=247)^a^	Incomplete (n=31)^b^
Age (years), median (SD)		25.0 (5.7)	29.0 (7.4)
Born in the United States, n (%)		210 (85.7)	14 (51.9)
**Race, n (%)**			
	White	165 (67.4)	11 (40.7)
	Latina/Hispanic	15 (6.1)	6 (22.2)
	Black/African American	25 (10.2)	7 (25.9)
	Asian	15 (6.1)	1 (3.7)
	More than one race	25 (10.2)	2 (7.4)
**Educational attainment, n (%)**			
	High school graduate/GED or less education	14 (5.7)	5 (18.5)
	Some college or 2-year degree	30 (12.3)	6 (22.2)
	4-year college graduate	94 (38.5)	7 (25.9)
	More than 4-year college degree	106 (43.4)	9 (33.3)
**Total annual household income, n (%)**			
	Below $25,000	54 (22.0)	4 (14.8)
	$25,000 to $49,999	63 (25.7)	7 (25.9)
	$50,000 to $74,999	42 (17.1)	2 (7.4)
	$75,000 to $99,999	19 (7.8)	1 (3.7)
	$100,000 or more	39 (15.9)	4 (14.8)
	Prefer not to answer	11 (4.5)	6 (22.2)
	Don't know	17 (4.5)	3 (11.1)

^a^Missing—born in the US: n=1 (0.4%); race: n=2 (0.8%); education: n=3 (1.2%); income: n=2 (0.8%).

^b^Missing—born in the US: n=4 (12.9%); race: n=4 (12.9%); education: n=4 (12.9%); income: n=4 (12.9%).

## Discussion

We determined the feasibility of a multimodal recruitment approach for a study of ovulation and menstruation health by comparing in-clinic, community-based, and internet-based recruitment activities. Among the recruitment modalities, the majority of women were recruited via the internet after encountering advertising materials informing them of the study website. The race and ethnicity of the cohort was notably similar to the US population [24]. Based on census data from 2018, the US population was comprised of 166,038,755 women which was 50.8% of the total population. Of those women, 60.3% were White, 17.8% were Latina or Hispanic, 12.9% were Black or African American, 5.9% were Asian, and 2.2% identified as being of more than one race [24].

The participant characteristics did differ by recruitment location, suggesting that multimodal recruitment is a feasible solution to increase the variation in participant characteristics including race, ethnicity, and health characteristics such as BMI, oral contraceptive use, and whether physician diagnosed. These different characteristics may reflect differences in the source population. For example, approximately 59% of the patients at BMC come from an underserved population [25], whereas the community event was held in Jamaica Plain, a neighborhood that has a 53.6% White population and a median household annual income of $55,861 [26]. While use of the internet usage demographics are reported as 92% of White, 86% of Hispanic, and 85% of Black individuals in the US [27], those accessing the study via the internet were recruited through flyers, email communications, and personal social media networks. The geographic distribution of the participants was notable for the majority in Massachusetts, with second-highest participation in Missouri, corresponding to the personal network of 1 research assistant.

Similarly, another study [28] was also successful in recruiting racially and ethnically diverse participants using a combination of 340 recruitment sites and digital campaigns. Among the core participants of that study [28], 51% were considered non-White, whereas participants in traditional reproductive health cohorts have been 83% Caucasian [29] and 89% White [14]. Furthermore, while participants from the original Framingham Heart Study were 100% White, racial diversity increased in subsequent studies with inclusion of 28% African American, 42% Hispanic American, and 24% Asian American participants within the Framingham-related Omni Cohort [30].

The completion rate among those who started our survey (247/278, 88.8%) was high. This completion rate is comparable to those of the Pregnancy Study Online [29], with 72% of enrolled participants completing the baseline survey, and the most recent Framingham Heart Study [31], with participants using an electronic survey (85% completion). It is possible that our survey was shorter than those used in those studies [29,31] and as such had a slightly higher completion rate. Furthermore, the Ovulation and Menstruation Health survey was designed for an 8th grade reading level and underwent cognitive and usability testing to facilitate question comprehension [32], potentially supporting a pleasant participant experience. While participants could skip any question, the rate of missing responses was low. Birthplace outside of the US was a key variable noted to be missing among a small proportion of women who did not complete the survey.

This study has several strengths. We achieved target recruitment in half the expected time, which may have been due to the appealing web-based study platform that included an educational cartoon featuring women from multiple backgrounds or the monetary incentive. Use of a cognitively tested survey designed for an 8th grade reading level may also have facilitated the high completion rate. The broad inclusion criteria also allowed for those with and without polycystic ovary syndrome to be included. Most importantly, the multimodal recruitment approach enabled us to recruit a more diverse cohort, which is comparable to those in other studies using a similar multimodal recruitment strategy for other health conditions [33].

However, the study also has some key limitations. While racial and ethnic diversity were improved compared to existing large epidemiologic cohorts, the proportion of Latina or Hispanic women was slightly lower than that in the US population, possible due to language access issues. We recently translated the Ovulation and Menstruation Health Platform for availability in Spanish language to test in future studies. Also, completion rate by those born outside the US may be improved by having the survey in the primary language of the participant. In the future, using paid advertisements may not only lead to high click-through rates to the study website, but may also provide for a unique opportunity to engage with specific targeted demographics depending on advertisement dissemination. For example, the Nurses’ Health Study 3 [17] sent recruitment postcards to minority-dense zip codes doubling the enrollment rate of African American and Hispanic women.

Furthermore, we considered whether bias in multimodal recruitment exists. We do not believe that multimodal recruitment increased the likelihood of selection bias since enrollment would need to be related to both the exposure and outcome under study for such a bias to occur [34-37]. The multimodal recruitment strategy increases the generalizability of the study and increases its ability to examine effect measure modification by racial and ethnic group by ensuring sufficient numbers in each category. Because differences exist in racial or ethnic-specific risks for a variety of health outcomes potentially due to structural racism and other inequities [38], a multiethnic cohort is needed to assess health outcomes subsequent to polycystic ovary syndrome diagnosis and to determine ideal windows for risk-reducing interventions.

Multimodal recruitment was feasible and established a more racially and ethnically diverse cohort for the study of ovulation and menstruation health than those of prior studies [14,28,29]. Samples from 3 different recruitment locations demonstrated variability in racial, ethnic, and other demographic and health-related features.

## Abbreviations

BMC: Boston Medical Center
BMI: body mass index
BUMC: Boston University Medical Campus
OB/GYN: Obstetrics and Gynecology
